# A New Perspective on the Origin of DNA Double-Strand Breaks and Its Implications for Ageing

**DOI:** 10.3390/genes12020163

**Published:** 2021-01-26

**Authors:** Bhabesh Kumar Tripathy, Kavita Pal, Snehal Shabrish, Indraneel Mittra

**Affiliations:** 1 Indian Institute of Science Education and Research (IISER), Tirupati 517507, India; bhabeshkumartripathy@students.iisertirupati.ac.in; 2 Tata Memorial Centre, Advanced Centre for Treatment, Research and Education in Cancer (ACTREC) and Homi Bhabha National Institute, Mumbai 410210, India; kavitapal80@gmail.com (K.P.); snehalrm@gmail.com (S.S.)

**Keywords:** free-radicals, cell-free chromatin, apoptosis, DNA damage, NHEJ, mosaicism, inflammation

## Abstract

It is estimated that 10–50 DNA double-strand breaks (DSBs) occur in a nucleated human cell per cell cycle. We reviewed the present state of knowledge and hypothesized that the currently accepted mechanisms cannot explain such high frequency of DSBs occurring daily under normal physiological conditions. We propose an alternative model that implicates illegitimate genomic integration into healthy cells of cell-free chromatin (cfCh) particles released from the billions of cells that die in the body every day. Repeated genomic integration of cfCh may have catastrophic consequences for the cell, such as DSBs, their faulty repair by nonhomologous end joining (NHEJ) followed by apoptosis with release of more cfCh which would integrate into genomes of surrounding cells. This can creates a vicious cycle of cfCh integration, DSBs, NHEJ, and more apoptosis, thereby providing a potential explanation as to why so many billions of cells die in the body on a daily basis. We also recount the recent observation that cfCh integration and the resulting DSBs activate inflammatory cytokines. This leads us to propose that concurrent DSBs and induction of inflammation occurring throughout life may be the underlying cause of ageing, degenerative disorders, and cancer. Finally, we discuss the prospect that agents that can inactivate/degrade cfCh may hold the key to making healthy ageing a realizable goal.

## 1. Introduction

What causes ageing and age-related diseases? These questions still do not have their final answers [1,2]. DNA double-strand breaks (DSBs) are considered to be the most dangerous among DNA lesions which increase with age [3,4,5], leading to cell death and cellular senescence [6]. Age-related increase in unrepaired DSBs may be a contributing cause of several degenerative disorders, such as Alzheimer’s disease [7], type 2 diabetes [8], cardiovascular disease [9], and cancer [10,11]. What causes DSBs? In this review, we propose that the currently accepted mechanisms may not fully explain the high frequency of DSBs that are seen daily in healthy cells and propose a new mechanism implicating cell-free chromatin (cfCh) particles that are released from billions of cells that die in the body every day. We go on to explain how this mechanism may help to throw new light on ageing and the various age-related disorders mentioned above.

DSBs in DNA occur when phosphodiester linkage between two adjacent nucleotides in each of the strands is cleaved simultaneously, and the sites of cleavage on opposite strands lie sufficiently close to each other. DSBs can be repaired by two pathways—homology dependent repair (HDR), one that is less frequent and less error-prone, and nonhomologous end joining (NHEJ), one that is more frequent and more error-prone [12]. In the HDR pathway, an intact homologous chromosome that shares sequence similarity with the damaged DNA is used as a template for repairing DSBs [12]. In contrast, during repair by NHEJ, the two ends are joined by DNA ligases without any need for a homologous sequence. Repair of DSBs by NHEJ can involve incorporation or deletion of random nucleotides into the damaged region, leading to modifications in the original DNA sequence [12]. DSBs constitute a potentially lethal form of DNA lesion and, if left unrepaired, or repaired imperfectly by NHEJ, can result in mutations, chromosomal rearrangements, and cell death. Defects in the cell’s response to DSBs have been linked to multiple disease conditions, such as neurological disorders [13], diabetes [14], and cancer [15].

It has been reported that 10–50 DSBs occur in a nucleated human cell per cell cycle [16]. However, human tissues and organs consist of both dividing, and non-dividing cells. Most epithelial cells have a rapid turnover rate and have a higher rate of DSBs and mutations which are related to development of cancer [17]. On the other hand, cells such as myocytes, adipocytes, and those of the skin and brain, undergo terminal differentiation and exist in a non-dividing state. The frequency of DSBs has been found to be lower in non-dividing cells [18,19]. However, DSBs do accumulate in quiescent cells and increase progressively with age [5]. For example, single-cell whole-genome sequencing has revealed that somatic single-nucleotide variants (sSNV) increase with age in human neuronal cells [20]. DSBs can disrupt neuronal activity and normal functioning of the nervous system and contribute to the development of neurodegenerative disease [21,22]. DSBs in DNA have been proposed to be caused by several exogenous agents, including ionizing radiation, ultraviolet (UV) light, and radiomimetic compounds, as well as by endogenous agents, including free radicals and DNA replication and transcription errors [23]. Radiation damage to cells can occur both by direct and indirect mechanisms [23]. In the direct mode of action, radiation excites nucleotides in the DNA directly, thereby introducing certain chemical modifications and consequently altering DNA structure. In the indirect mode of action, radiation splits-up water molecules in the cytoplasm and other organic molecules in the cell resulting in formation of free radicals, like hydroxyl (OH·) and peroxyl (ROO·) radicals, which have the potential to induce DSBs. However, significant levels of radiation are not normally present in the environment to explain high levels of DSBs. For example, it is reported that natural background radiation that a human is exposed to on a daily basis is ≈0.01 mSv [18]. This value varies across the Earth, but, in general, the impact of radiation on daily introduced DSBs inside our cells is very small. It is well established that ionizing radiation produces 25–40 DSBs per diploid cell per gray [24]. Hence, natural sources of radiation cannot be the reason for multiple DSBs that appear in our cells daily.

Free radicals are normally generated as by-products of cellular metabolism occurring in mitochondria [25], peroxisomes [26], and the endoplasmic reticulum (ER) [27]. At high levels, free radicals can induce oxidative and nitrosative stress that can potentially damage biomolecules, including proteins [28], lipids [29], and DNA [30]. Hydroxyl (OH·) radicals, one of the most damaging free radicals, react with nucleotide bases and deoxyribose sugars and introduce chemical modifications in them, including the formation of abasic sites and generation of single-strand breaks (SSBs) in DNA [31]. These base modifications and single-strand DNA damage are repaired via the base excision repair pathway [32]. The latter proceeds through the removal of damaged bases, formation of SSBs, and further processing to repair and seal off the damage [32]. It has been shown in instances where two SSBs are present on alternate strands in close proximity, they can be spontaneously converted into DSBs during their repair process [16]. However, hydroxyl radicals have a very short in vivo half-life of approximately 10^−9^ s [33]; and, once they are produced, they can react only with molecules close to their site of production. Hence, they can, at best, attack mitochondrial DNA but, in all probability, do not make it to the genomic DNA. H_2_O_2_, another toxic species, is said to indirectly damage DNA by producing hydroxyl radicals in presence of transition metal ions (Fenton reaction) or by reacting with superoxide radicals (Haber-Weiss reaction) [34]. However, the availability of transition metal ions in sufficient amounts near the genomic DNA to take part in the Fenton reaction is questionable [35]. It is also not evident if there is ever enough H_2_O_2_ to escape the antioxidant defense system of the cells to damage the genomic DNA [36]. Singlet oxygen (^1^O_2_) is another free radical that can induce DNA damage and has been shown to react selectively with guanine moiety in nucleosides and introduce SSBs in plasmid DNA [37]. Currently, there is no evidence of it (^1^O_2_) inducing DSBs in the genomic DNA.

Other endogenous mechanisms by which DSBs may occur are DNA replication and transcription errors. SSBs and DNA crosslinking due to covalent linkage between two nucleotides either within the same strand or between two opposite strands of DNA, referred to as DNA intra-strand and inter-strand crosslinks, respectively, or due to covalent linkage between DNA and proteins can introduce lesions in DNA and interfere in DNA replication. During the latter, DNA polymerase may stall when it encounters these lesions, thereby disrupting the replication fork, leading to formation of DSBs [38,39,40]. However, this hypothesis may not be applicable to neuronal cells that do not divide, and especially since DSBs in brain increase progressively with age and are linked with neurodegenerative disorders, like Alzheimer’s disease [41]. Another hypothesis is the formation of DSBs from R loops formed during transcription [42]. R loops are triple stranded nucleic acid structures consisting of a DNA:RNA hybrid structure along with the displaced DNA strand. They are mostly formed during the elongation step of transcription when a portion of nascent RNA transcript re-anneals to its DNA complement. But whether or how R loops cause DSBs is not known [42].

The above review of the literature leads us to conclude that the currently accepted mechanisms cannot explain the high frequency of DSBs that have been reported to occur daily under normal physiological conditions and that new mechanisms to explain DSBs are needed. We provide below one such new mechanism which involves DSBs inflicted by genomic integration of cell-free chromatin (cfCh) particles released from dying cells.

## 2. Cell-Free Chromatin: A Continuously Arising Trigger for DSBs

Several hundred billion to a trillion cells, largely of haematogenous origin, die in the adult human body daily due to normal physiology, and a similar number is regenerated by mitosis to maintain homeostasis [43]. The daily turnover of granulocytes has been reported to be of the order of 120 × 10^9^ cells, that of erythrocytes to be 200 × 10^9^ cells, of platelets to be 150 × 10^9^ cells and of lymphocytes to be 20 × 10^9^ cells [43]. This high rate of cell death on a daily basis occurs primarily by apoptosis which is characterized by condensation and fragmentation of chromatin mediated by endogenous nucleases [44]. Fragmentation and inter-nucleosomal cleavage of DNA leads to formation of oligo-nucleosomes with multiples of 180–200 base pairs [45,46]. A proportion of the nucleosomal fragments (cell-free chromatin, cfCh) generated following cell death finds entry into the extra-cellular compartments of the body, including into the circulation [47,48,49]. Increasing cfCh levels are positively correlated with age [50,51], and elevated blood levels have been reported in many acute and chronic human disorders, including cancer [47,52,53,54,55,56,57,58,59,60,61]. cfCh is cleared by the body by several mechanisms, which include: (1) phagocytotic clearance by macrophages [62]; (2) degradation of DNA component of cfCh by DNase I present in circulation [63]; and (3) continuous clearance by the liver resulting in a turnover half-life of cfCh in circulation of 10–15 min [64,65].

### 2.1. cfCh Particles in Circulation Inflict DSBs in Healthy Cells by Integrating into Their Genomes

Chromatinized genes generated by in vitro reconstitution of DNA with histones are known to be efficiently taken up by cells followed by their genomic integration [66]. Although the first report of cfCh in human circulation appeared in 1990 [67], whether they have any patho-physiological role to play in the host has only recently been addressed [51,68]. cfCh particles were successfully isolated from sera of cancer patients and healthy volunteers, which upon electron microscopy (EM) examination appeared as particles of heterogeneous sizes (~10 nm > 1000 nm) having a beads-on-a-string appearance typical of chromatin. When cfCh particles that were fluorescently dually labeled in their DNA and histones were applied to mouse fibroblast cells, numerous dually labeled fluorescent signals were detected in nuclei of the treated cells with a maximum nuclear uptake reaching at 6 h. Nuclear uptake was rapidly followed by their association with host cell chromosomes which led to intense activation of a DNA damage repair response (DDR), which facilitated their incorporation into the host cell genome via a unique mechanism described later in this article. The up-regulated DDR proteins included H2AX, ATM, ATR, MDC-1, P-p53, P-p21, GADD-34, Nibrin, Rad50, MRE-11, DNA-PKcs, and DNA ligase IV. In addition, up-regulated were proteins of the apoptotic pathway, namely JC-1, cytochrome-C, and caspase 3, indicating that DSBs were likely to result in apoptosis of many affected cells [68].

Multiple lines of evidence were advanced to demonstrate that cfCh had truthfully integrated into the genomes of the recipient cells. Fluorescent in situ hybridization (FISH) using human-specific whole genomic and pan-centromeric probes showed multiple human DNA signals in two single cell clones that had been developed from the mouse fibroblast cells that had been treated with cfCh isolated from cancer patients. Next, generation genomic sequencing of the single-cell clones detected tens of thousands of human reads in the mouse cells, while PCR amplification of DNA from them identified multiple human Alu sequences [68].

When cfCh particles isolated from cancer patients were injected intravenously into mice, numerous human DNA signals were detected in nuclei of cells of their vital organs upon analysis by FISH. That genomic integration of cfCh particles were indeed responsible for DSBs was confirmed by detection of precise co-localization of human FISH signals with those of γ-H2AX in brain cells of mice [68]. Extensive activation of H2AX was also seen in cells of all other vital organs by immunofluorescence analysis. Significantly, cfCh from cancer patients were invariably found to be more active in inducing DSBs than those isolated from healthy volunteers, suggesting a possible role of the former in pathogenesis of cancer. Finally, DSBs induced by cfCh were abrogated both in vitro and in vivo when concurrently treated with cfCh inactivating agents, namely anti-histone antibody-complexed nanoparticles (CNPs) and/or DNase I [68,69].

### 2.2. cfCh Particles Released Locally from Dying Cells Inflict DSBs in Bystander Cells

Two recent publications have reported that cfCh particles released locally from dying cells can be freely ingested by bystander healthy cells. This was demonstrated by dually labeling Jurkat cells in their DNA and histones with BrdU and CellLight^®^ Histone 2B GFP respectively, followed by treating the labeled cells with ionizing radiation (15 Gy). When the fluorescently labeled dying Jurkat cells were co-cultured with mouse fibroblast cells and examined by confocal microscopy, numerous dually labeled fluorescent particles that were released from the dying Jurkat cells were found to have been up-taken by the co-cultured cells to accumulate in their nuclei within 24 h [70,71]. Two single-cell clones were prepared from the co-cultured mouse fibroblast cells and subjected to whole genome sequencing and bioinformatics analyses which detected 209 human Alu elements represented by 15 unique human Alu families when data derived from the two clones were combined. Genomic integration of human cfCh particles led to extensive DSBs and chromosomal aberrations in the mouse cells [71]. Delivery of focused micro-beam irradiation to the umbilical region of mice resulted in intense activation of H2AX in brain cells, indicating that cfCh released from radiation-induced dying cells can travel to distant organs via the bloodstream to inflict DSBs.

### 2.3. cfCh Particles Released from Dying CTCs Inflict DSBs in Distant Organs

It is well established that circulating tumor cells (CTCs) undergo extensive cell death upon reaching target organs [72,73]. In experiments in which viable mouse melanoma cells that had been fluorescently pre-labeled were injected intravenously into mice, numerous fluorescent cfCh signals were detected in the nuclei of their vital organs. Immune staining analysis showed precise co-localization of fluorescent cfCh signals with those of γ-H2AX [70]. This finding indicated that cfCh particles released from CTCs that die upon reaching distant organs can integrate into genomes of target cells to inflict DSBs.

## 3. cfCh Particles Integrate into Genomes Via a Unique Mechanism to Inflict DSBs

**Figure 1:**
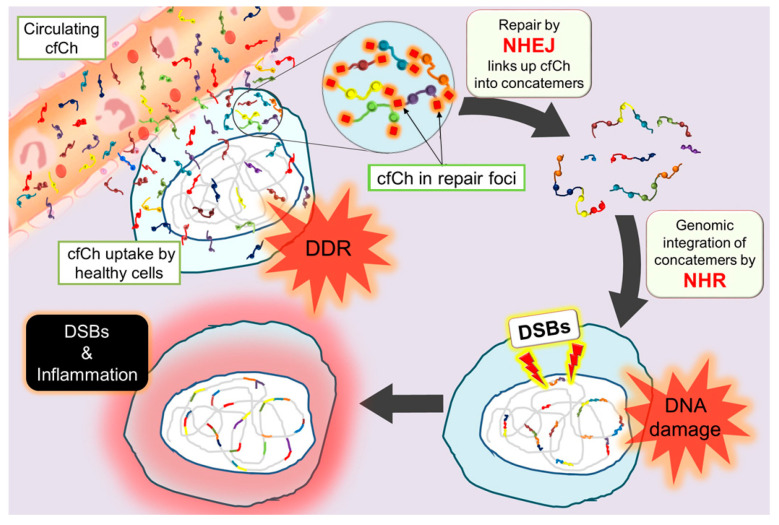
Schematic representation of the unique mechanism by which cell-free chromatin (cfCh) particles integrate into the genome to inflict double-strand breaks (DSBs) and to activate an inflammatory response. NHEJ: nonhomologous end-joining; NHR: nonhomologous recombination. It is to be noted that in this model damage repair response (DDR) precedes DNA damage.

A unique model by which cfCh particles integrate into genomes of healthy cells has been proposed wherein the sequence of DSBs and activation of DDR are reversed (Figure 1) [68,74].

In the classical model, DDR is activated after DSBs have been induced by damaging agents, such as ionizing radiation, UV, and radiomimetic chemicals. According to the proposed model, on the other hand, DDR is prematurely activated prior to DNA damage. Internalized cfCh particles ‘deceive’ the cell into perceiving them as fragments of its own chromosomes with DSBs at both broken ends, leading the cell to mount a DDR/repair response well before any DNA damage has actually occurred. The DDR activates various repair proteins, especially, DNA PK-cs and DNA ligase IV, which link up the numerous disparate intra-cellular cfCh fragments into concatamers of variable sizes containing a mosaic of discontinuous DNA segments that form new substrates for integration into the host cell genomes. Genomic integration of concatamers occurs via nonhomologues recombination with potentially catastrophic consequences, such as mutations, rearrangements, and various other modifications in the genome. Thus, according to the new model, genomic integration of extraneous cfCh concatamers comprising of a mosaic of DNA segments, which might also include centromeric sequences, may have far-reaching consequences beyond the induction of DSBs. These consequences are likely to include formation of extensive genetic rearrangements and mosaicism, genomic instability, and potentially oncogenic transformation and/or apoptosis of the affected cell [68,74]. Recent reports suggest that genomic integration of cfCh or its concatamers also activate inflammatory cytokines, as discussed later in this article.

## 4. cfCh Particles Propel a Vicious Cycle of DSBs, Apoptosis and more DSBs

In this section, we try to analyze why so many billions of cells die in the body every day? Clearly, DSBs inflicted by integration of cfCh concatamers and their repair by NHEJ may often prove too toxic an assault on the genome which may cause apoptosis of the affected cell. The latter would lead to release of more cfCh particles which may integrate into surrounding bystander cells to trigger a vicious cycle of DSBs followed by imperfect repair by NHEJ and further rounds of apoptosis (Graphical abstract). Perpetuation of this vicious cycle is likely to generate more complex mosaic concatamers which upon genomic integration will progressively escalate further rounds of DSBs, apoptosis and mosaicism of the genome. Cellular apoptosis will also release cfCh particles into the circulation and perpetuate similar vicious cycles in cells of distant organs, leading to systemic mosaicism. This model may thus help to throw light on the question as to why the somatic genome becomes progressively unstable and mosaic with increasing age [4]. The model also reflects the scale and consequences of day-to-day apoptosis of billions of cells and the vicious cycle that they create to generate progressively increasing mosaicism and genetic instability throughout all cells of the body which would increase with age.

## 5. cfCh Integration and DSBs Activate Inflammation

Emerging evidence suggests that DSBs inflicted by cfCh integration into the genome can lead to activation of inflammatory cytokines [70,71,75,76]. Co-cultivation of irradiated dying human cancer cells with mouse fibroblasts not only resulted in activation of H2AX, as described above but also to that of multiple inflammatory cytokines in the fibroblast cells. These included the transcription factor NFκB, as well as other cytokines, such as IL-6, TNF-α, and IFN-γ. The inflammatory cytokines were up-regulated concurrently, reaching a maximum at ~6 h, which coincided with the point of maximum activation of H2AX. The interrelationship between cfCh-induced DSBs and inflammation was supported by microarray studies wherein the timing of up-regulation of multiple pathways related to inflammation coincided with those associated with cell cycle and DNA damage. Further confirmation of an association was provided by the finding that treatment with cfCh inactivating agents not only prevented activation of H2AX, but also that of NFκB and other inflammatory cytokines [70]. Intravenous injection of dying cancer cells into mice led to activation of both H2AX and inflammatory cytokines in cells of target organs. Significantly, fluorescent signals of NFκB were found to co-localize with those of γ-H2AX signals. NFκB under normal conditions is known to reside in the cytoplasm; but the violent event of cfCh integration into the genome apparently activates it to translocate to the nucleus particularly to the sites of cfCh integration to co-localize with γ-H2AX. These findings have given rise to the suggestion that inflammation may be a direct consequence of cfCh-induced DSBs [75,76]. Such a proposal is supported by the observation that a direct positive correlation exists between serum levels of cfCh and inflammatory cytokines in healthy human volunteers [51].

Recently, there have been a number of publications on DNA sensing GMP-AMP synthase-stimulator of interferon genes (cGAS-STING) pathway activation in response to accumulation of DNA in the cytoplasm [77,78,79,80]. The latter can occur either following entry of microbes into the cell or due to genomic stress [77]. These, in turn, activate pro-inflammatory cytokines and trigger an innate immune response [77,78,79,81]. Activation of cGAS-STING pathway in response to cytoplasmic chromatin fragments (CCF) has also been reported [82,83,84]. Presence of DNA or CCF in the cytoplasm leads to activation of two downstream pathways, namely the IRF3 pathway, resulting in production of type I interferon, and second, the NFκB pathway, resulting in production of multiple pro-inflammatory cytokines [77].

Can the uptaken extraneous cfCh particles activate cGAS-STING? Considering our findings discussed above, the possibility that presence of cfCh particles in the cytoplasm induce NFκB via the cGAS-STING pathway cannot be excluded. However, if it were to be so, an explanation would be needed for the finding of co-localized fluorescent signals of NFκB and γ-H2AX precisely at sites of cfCh integration. The latter would require cytoplasmic NFκB to undergo activation due to cfCh integration followed by its translocation precisely at the sites of cfCh integration. Nonetheless, further studies are required to investigate whether cfCh activation of NFκB occurs via the cGAS-STING pathway or is a direct consequence of DSBs following cfCh integration?

## 6. cfCh-Induced DSBs, Somatic Mosaicism, and Inflammation in the Etiology of Ageing, Chronic Diseases, and Cancer

Rapid advances in next-generation sequencing are revealing that post-zygotic genomes of the same individuals are remarkably heterogeneous. Genome of one cell of the same individual can differ from another in terms of single nucleotide polymorphisms, copy-number variations, insertions, deletions, inversions, translocations, and other structural rearrangements and chromosomal variations [85,86]. Genomic mosaicism increases with age and has been shown to be associated with ageing and age-related disorders, such as Alzheimer’s disease [7], type 2 diabetes [8], cardiovascular disease [9], and cancer [10,11]. Although, several studies have suggested DSBs to be a primary underlying cause of ageing [3,4,5], the mechanism(s) by which DSBs are inflicted is not fully understood [5]. More efficient genome maintenance mechanisms in long-lived compared to short-lived organisms have been attributed to more timely and effective expression of genes encoding DDR in the former [87]. Emerging evidence has also implicated defects in DDR signaling to be a key mechanism underlying DNA damage, cell senescence and ageing [88]. DDR activation in senescent cells promotes acquisition of a pro-inflammatory secretory phenotype (SASP), which further elicits DDR and SASP activation in bystander cells, thereby creating a pro-inflammatory environment at the local, and eventually at the systemic level. Therefore, accumulation of cells with an activated DDR probably fuels “inflamm-ageing” that predisposes to development of the many age-related disorders [88].

Our proposal of genomic integration of cfCh particles and/or their concatamers resulting in DSBs followed by imperfect repair by NHEJ occurring throughout life may help to provide an explanation not only for the progressively increasing mosaicism of the ageing genome [4] but also for the multiple degenerative disorders that are associated with ageing. The severe genomic damage will also bring about considerable changes in the epigenome, which may further contribute to the development of ageing-related disease phenotypes [89]. As discussed above, DNA and chromosomal damage will activate an inflammatory response which introduces a new facet to the underlying complexity that contributes to occurrence of the multiple disease conditions [90,91,92,93,94]. Thus, the triple pathologies of DSBs, somatic mosaicism and inflammation, brought about by a common event of cfCh integration into the genome, may provide a unifying model for our understanding of the etiologies of the many chronic disorders that are associated with ageing.

## 7. Is it cfCh or cfDNA that Cause DSBs?

Cell-free DNA (cfDNA) and cfCh have been shown to have distinct biological activities [95]. Differences exist in cytotoxic activities that are induced individually by cfDNA and when cfDNA is complexed with histones in the form of nucleosomes (cfCh) [95]. In real life, chromosomal condensation and fragmentation following apoptotic cell death results in release of mono- and poly-nucleosomes, and not free DNA [96]. The presence of nucleosomes (cfCh particles) in serum and/or plasma can be directly demonstrated by an ELISA assay that uses antibodies to both DNA and histones [97]. On the other hand, demonstration of cfDNA requires Proteinase-K treatment prior to DNA extraction. Therefore, the possibility that the isolated cfDNA is in effect derived from circulating cfCh cannot be excluded. This is further supported by the several reports of a strong positive correlation between circulating cfCh and cfDNA [98]. Therefore, the question remains as to whether naked DNA exists in circulation [95]? Our recent study of endotoxin-induced sepsis in a mouse model, helps to put this uncertainty to rest by demonstrating that, lipopolysaccharide (LPS)-induced dying host cells release cfCh particles and not free DNA [99]. Although there is much current interest in cfDNA as a biomarker in cancer diagnostics and therapy response [100], it is far from clear whether naked DNA circulates as such in the blood as a natural biological molecule. Thus, the biological agent responsible for DSBs is probably cfCh and not cfDNA.

## 8. Conclusions

In this article, we have summarized the currently accepted mechanisms that underlie DSBs and conclude that none of them can fully explain the high frequency of DSBs that occur in nucleated human cells per cell cycle on a daily basis (10–50/cell) under normal physiological conditions. We have proposed an alternative mechanism which involves illegitimate genomic integration into healthy cells of cfCh particles released from the billions of cells that die in the body every day. Such repeated genomic integration of cfCh particles and repair by NHEJ on a daily basis may account for the high number of DSBs that have been reported in the literature. We have also proposed that premature activation of DDR, leading to intra-cellular ligation of disparate cfCh segments with formation of large and mosaic concatamers may inflict catastrophic damage to the genome upon their genomic integration by nonhomologous recombination. The latter might cause death of the affected cells with release of cfCh particles, leading to a vicious cycle of further cell death propelled by release of more cfCh particles, thereby providing a possible explanation as to why so many billions of cells die in the body on a daily basis. We have also discussed the novel finding that cfCh integration and DSBs result in activation of inflammatory cytokines, which has led us to propose that concurrent induction of DSBs, genomic instability and inflammation occurring throughout life may be the underlying cause of ageing, the various degenerative disorders, and cancer.

## 9. Future Prospects

Our proposed novel mechanism of induction of DSBs leads to the logical inference that inactivation of cfCh particles may have health benefits, including retardation of ageing. In this context, we have reported that several cfCh inactivating agents have therapeutic effects [71,99,101]. These agents have included (1) nanoparticles complexed with anti-histone antibodies which inactivate cfCh particles by binding to histones [69]; (2) DNase I which degrades the DNA component of cfCh particles, thereby depriving it of its damaging effects; and (3) a novel combination of the nutraceuticals resveratrol and metallic copper, which degrades cfCh particles via the medium of free radicals [102]. We have reported that all three cfCh inactivating/degrading agents can prevent pathologies associated with multiple acute conditions, all of which involve cfCh-induced DSBs and inflammation. For example, these agents can ameliorate toxic side-effects of chemotherapy [101], radiation therapy [71], and sepsis-related cytokine storm and fatality in endotoxin-treated mice [99].

Of the three cfCh inactivating agents, the combination of the nutraceuticals Resveratrol and Copper holds the maximum promise. Resveratrol is a plant polyphenol found in skin of red grapes and berries which has been extensively researched for its antioxidant properties [103]. Metallic copper, likewise, has been widely tested in pre-clinical studies for its potential health benefits [104]. We have discovered that when Resveratrol and Copper are combined, a remarkable reaction is triggered [71,99,101]. Resveratrol has the ability to reduce Cu (II) to Cu (I) to generate highly unstable free radicals, which can damage cellular organelles, proteins, lipids, DNA, and RNA [28,29,30]. We have used an oral combination of small quantities of Resveratrol and Copper (R-Cu) in our pre-clinical studies to demonstrate that free radicals thus generated can degrade cfCh particles, leading to prevention of several pathological conditions associated with cytokine storm [71,99,101]. If our preclinical results are replicated in human trials, R-Cu may prove to be an ideal agent that might help to retard ageing and age-related degenerative disorders. Clinical trials would be required to investigate if R-Cu could make healthy ageing a realizable goal.

## Data Availability

Not applicable.
